# Nurse-Led Secondary Prevention After Acute Coronary Syndrome: Bridging the Gender Gap in Cardiovascular Outcomes—A Sub-Analysis of the BEAT-HF Study

**DOI:** 10.3390/jcdd13020102

**Published:** 2026-02-21

**Authors:** Oona Meroño Dueñas, Mar Iraculis Sanchez, Marc Llagostera Martin, Marta Ruiz Muñoz, Marta Gomez Cuba, Laia Alcober Morte, Natalia López Fernández, Guillem Cirera Salleras, Adrian Ricarte Marin, Maria Soler Cera, Alberto Garay Melero, Gemma Simo Cubel, Joan Antoni Gomez Hospital, Cristina Capdevila Aguilera, Josep Comin Colet

**Affiliations:** 1 Department of Cardiology, Bellvitge University Hospital—Bellvitge Biomedical Research Institute (IDIBELL), 08907 L’Hospitalet de Llobregat, Spain; mllagosteram@bellvitgehospital.cat (M.L.M.);; 2 Bioheart Group, Cardiovascular, Respiratory and Systemic Diseases and Cellular Aging Program, Bellvitge Biomedical Research Institute (IDIBELL), 08908 L’Hospitalet de Llobregat, Spain; jcomin@bellvitgehospital.cat; 3 School of Medicine, Universitat de Vic-Central de Catalunya, 08500 Vic, Spain; 4 Ciber Cardiovascular Group (CIBER-CV), Instituto Salud Carlos III, 28029 Madrid, Spain; 5 Department of Physical Medicine and Rehabilitation, Bellvitge University Hospital, 08907 L’Hospitalet de Llobregat, Spain; martagomez@bellvitgehospital.cat; 6 Department of Clinical Sciences, School of Medicine, Universitat de Barcelona (UB), 08036 Barcelona, Spain; 7 Primary Care Management and The Delta and Baix Llobregat Community, GAPiC Delta, Institut Catala de la Salut, 08036 Barcelona, Spain; 8 Evaluation Unit, Department of Data Management, Bellvitge University Hospital, 08907 L’Hospitalet de Llobregat, Spainguillemcirera@bellvitgehospital.cat (G.C.S.); aricarte@bellvitgehospital.cat (A.R.M.); msolerce@bellvitgehospital.cat (M.S.C.); 9 Department of Gerence, Bellvitge University Hospital, 08907 L’Hospitalet de Llobregat, Spain; 10 Cardiology Department, Bellvitge University Hospital, 08907 L’Hospitalet de Llobregat, Spain

**Keywords:** cardiac rehabilitation, nurse-led care, gender disparities, secondary prevention, acute coronary syndrome, therapeutic exercise, health equity

## Abstract

**Background:** Despite advances in the management of acute coronary syndrome (ACS), women continue to experience higher long-term mortality and lower access to secondary prevention compared with men. **Objective:** This study aimed to assess whether a universally inclusive, nurse-led secondary prevention program implemented at a University Hospital improved post-ACS outcomes and reduced gender disparities in risk factor control and mortality. **Methods:** This retrospective, observational study compared two cohorts of ACS survivors discharged from Bellvitge University Hospital: a pre-intervention cohort (2018) and a post-intervention cohort (2022). The nurse-led program included universal enrollment of all ACS patients, early follow-up, pharmacological optimization, therapeutic exercise, lifestyle counseling, and coordination with primary care. Outcomes included lipid and glycemic control and 18-month mortality, stratified by sex. Results: A total of 409 patients were included (2018: n = 200; 2022: n = 209), of whom 130 were women. Women were older and had more comorbidities. Post-program implementation, the proportion of patients without post-discharge blood testing dropped from >50% to <17% in both sexes. Lipid and glycemic control improved significantly at both early (1–4 months) and late (9–18 months) follow-up. Early differences favoring men disappeared by 18 months. Mortality decreased by 27.5% in men and 47.6% in women, representing a significantly greater relative reduction among women (*p* = 0.0001). **Conclusions:** A structured, nurse-led secondary prevention program with systematic inclusion improved clinical outcomes and significantly narrowed the gender gap in cardiovascular mortality. These findings demonstrate that equitable, protocolized care led by advanced practice nurses can reduce systemic inequities in cardiovascular health.

## 1. Introduction

Cardiovascular disease (CVD) remains the leading cause of death worldwide, responsible for over 18 million deaths annually [1]. Although advances in acute coronary syndrome (ACS) management—such as reperfusion therapy and pharmacological optimization—have markedly improved in-hospital survival, long-term outcomes remain suboptimal. Nearly one in five patients experiences recurrent events or death within two years after ACS [2,3].

Secondary prevention is essential to address this residual risk. Cardiac rehabilitation (CR), combining therapeutic exercise, pharmacological optimization, psychosocial support, and lifestyle counseling, has proven to reduce morbidity and mortality significantly [4]. Yet, participation in CR remains alarmingly low, particularly among women [5].

Women presenting with acute coronary syndrome (ACS) are typically older and exhibit a higher prevalence of comorbidities—such as diabetes, hypertension, and obesity—alongside atypical or less-recognized symptoms that frequently result in diagnostic or therapeutic delays [6,7]. These clinical challenges are compounded by systemic inequities, as women are statistically less likely to receive guideline-directed medical therapy or be referred to structured cardiac rehabilitation programs [8,9,10]. Furthermore, biological factors such as smaller coronary artery diameters and the limited recognition of female-specific risk factors such as pregnancy-related complications, premature menopause, and the loss of estrogen-mediated cardioprotection following menopause and autoimmune disorders [11,12,13] exacerbate their susceptibility to ischemia and contribute to a higher residual risk of recurrent events. Collectively, these multifactorial gaps in care and physiology underpin the poorer long-term prognosis and higher cardiovascular mortality rates consistently observed in female populations compared to their male counterparts [14,15].

Addressing these disparities requires structural solutions that ensure equity by design. In late 2021, Bellvitge University Hospital (Barcelona, Spain) implemented a comprehensive secondary prevention program led by an advanced practice nurse (APN) specializing in cardiovascular care. Unlike traditional referral-based models, this program adopts a principle of universal inclusion, whereby every patient admitted with ACS is automatically enrolled into structured follow-up, irrespective of sex, age, or comorbidities.

The program integrates hospital and primary care, ensures early post-discharge review, and systematically includes therapeutic exercise as part of cardiac rehabilitation for all eligible patients. This study evaluated the program’s impact on cardiovascular mortality and control of modifiable risk factors, emphasizing gender-based differences and the role of advanced nursing leadership in improving equity and outcomes.

## 2. Methods

### 2.1. Study Design and Setting

A retrospective, observational, analytical study was conducted using data from the BEAT-HF (Big data & real-world Evidence to assess the healthcare and health outcomes burden of Acute coronary syndromes complicaTed with Heart Failure.) project.

**Figure 1:**
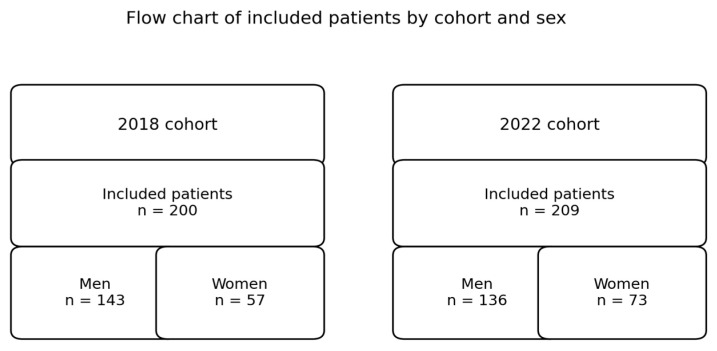
Flow chart of included patients by cohort and sex.

The study population includes all patients assigned to the healthcare area covered by Bellvitge University Hospital—a tertiary referral center serving approximately 1 million residents—which includes L’Hospitalet de Llobregat and El Prat de Llobregat, who were diagnosed with ACS and discharged during two reference years (Figure 1):

Pre-intervention cohort (2018): before the implementation of the structured program.

Post-intervention cohort (2022): after the implementation of the nurse-led program.

All consecutive ACS (STEMI, NSTEMI, or unstable angina) patients discharged alive in 2018 and 2022 were included. Exclusion criteria were in-hospital death, missing follow-up data, or non-residency in the hospital’s reference area.

### 2.2. Program Description

The secondary prevention program was implemented in December 2021. The program was designed and implemented by a multidisciplinary group of cardiologists, nurses, consultants in rehabilitation medicine, primary care physicians, clinical dietitians, and physiotherapists, and it is coordinated by an advanced practice nurse (APN).

Key components included the following:

1. Universal/systematic inclusion: all patients discharged after ACS were automatically enrolled.

2. Standardized pathways: early follow-up within 6–8 weeks post-discharge, medication optimization, guideline-based management, and primary care coordination.

3. Therapeutic exercise: systematic referral to a structured exercise program, implemented from April 2022, unless contraindicated.

4. Lifestyle and psychosocial counseling: individualized dietary and behavioral interventions.

### 2.3. Variables of Interest

The study included sociodemographic, clinical, and episode-related variables. Sociodemographic variables included sex and age at the time of the index acute coronary syndrome (ACS).

Clinical variables comprised cardiovascular risk factors and comorbidities recorded at hospital discharge, including diabetes mellitus, arterial hypertension, dyslipidemia, active smoking, obesity, chronic kidney disease, prior stroke, atrial fibrillation or flutter, previous coronary events, and obstructive sleep apnea.

Episode-related variables included the type of ACS, categorized as ST-elevation myocardial infarction (STEMI), non-ST-elevation myocardial infarction (NSTEMI), or other.

The primary outcome was cardiovascular mortality within 18 months after hospital discharge. Secondary outcomes included the control of LDL cholesterol and HbA1c at two predefined follow-up timepoints: (1) between 30 and 120 days after discharge and (2) between 9 and 18 months after discharge.

Patients without a recorded laboratory result within the specified time windows were considered uncontrolled, following an intention-to-monitor approach. Lipid control was assessed in accordance with current clinical practice guidelines. Specifically, we calculated the proportion of patients achieving LDL cholesterol levels <55 mg/dL and <70 mg/dL, as well as those achieving non-HDL cholesterol levels <85 mg/dL and <100 mg/dL.

To better reflect guideline-based risk stratification, two composite variables were created following the 2021 recommendations of the European Society of Cardiology (ESC). STEP 1 lipid control: defined as achieving LDL-c < 70 mg/dL and/or non-HDL cholesterol < 100 mg/dL. STEP 2 lipid control: defined as achieving LDL-c < 55 mg/dL and/or non-HDL cholesterol < 85 mg/dL. These composite outcomes allowed us to evaluate the degree of lipid control according to both standard and intensified therapeutic goals for very high-risk patients, such as those with ACS.

In patients with diabetes, glycemic control was defined as HbA1c < 7%, in accordance with guideline recommendations.

### 2.4. Pharmacological Intensification

Pharmacological intensification differed between cohorts due to the implementation of a structured secondary prevention program in 2022.

In the pre-program cohort (2018), lipid-lowering treatment intensification was left to the discretion of the treating cardiologist during routine outpatient follow-up, without a standardized protocol for laboratory monitoring or treatment escalation.

In contrast, following the implementation of the nurse-led secondary prevention program in 2022, lipid management was protocolized and coordinated before hospital discharge. The first lipid profile was systematically scheduled at 6 weeks after discharge. If LDL-cholesterol targets were not achieved, pharmacological treatment was intensified, and lipid levels were reassessed after an additional 8 weeks. This stepwise approach was repeated until guideline-recommended LDL-cholesterol targets were reached. Treatment intensification followed a predefined strategy, including up-titration of statins to the maximum tolerated dose, addition of ezetimibe when not previously prescribed, and initiation of PCSK9 inhibitors in patients fulfilling regional prescription and reimbursement criteria (LDL-cholesterol > 100 mg/dL in patients with statin intolerance or despite maximally tolerated oral lipid-lowering therapy).

### 2.5. Data Collection

Demographic (sex, age), clinical (risk factors, comorbidities), and episode-related variables (ACS type) were extracted from Business Intelligence Dashboard (BID), which aggregates clinical data, laboratory results, and mortality records, allowing sex-disaggregated analyses. This BID was designed within the framework of the DAIPO project (“Noves capacitats i Transformació Digital en l’atenció integrada a pacients amb patologies complexes”) and Novartis collaboration. Statistical analyses were stratified by sex.

### 2.6. Outcomes and Statistical Analysis

The primary outcome was 18-month cardiovascular mortality, and secondary outcomes were lipid and glycemic control at early (1–4 months) and late (9–18 months) follow-up, based on ESC STEP 1 and STEP 2 lipid targets.

Descriptive statistics summarized the baseline characteristics. Inferential analyses were used to assess changes between the two cohorts (2018 vs. 2022) and sex-based differences. Categorical variables were compared using the chi-square test or Fisher’s exact test, as appropriate. Continuous variables were compared using the non-parametric Mann–Whitney U test.

Survival analysis was conducted using Cox proportional hazards regression models to estimate hazard ratios (HRs) for cardiovascular mortality. The proportional hazards assumption was visually assessed by inspecting the survival curves; since the curves did not intersect, the assumption was considered met. Kaplan–Meier survival curves were also generated to describe time-to-event outcomes (cardiovascular death within 18 months), and the log-rank test was used to compare survival distributions between groups.

The proportions of patients with controlled LDL cholesterol and HbA1c levels were compared between cohorts and by sex using chi-square or Fisher’s exact tests, as appropriate. The percentage of patients who underwent laboratory testing at each timepoint (30–120 days and 9–18 months) was also described and compared between cohorts and sexes using chi-square tests. Multivariable logistic regression identified predictors of achieving STEP 2 lipid control. All analyses were stratified by sex.

A *p*-value < 0.05 was considered statistically significant.

### 2.7. Ethical Considerations

This study is a subanalysis of the project titled “Big data and real-world evidence to assess the healthcare and health outcomes burden of acute coronary syndromes complicated with heart failure: the BEAT-HF Study” (code: BEAT-HF study). The ethical approval covers all procedures involved in data management and patient protection. All procedures were conducted in accordance with the ethical principles of the most recent version of the Declaration of Helsinki (75th World Medical Association General Assembly, Riga, 2024), as well as with the principles of the ICH Guideline for Good Clinical Practice (ICH-GCP, 2025). All data were retrospectively obtained from anonymized clinical records.

## 3. Results

### 3.1. Study Population

The baseline characteristics of the study population were analyzed in two complementary ways to provide a comprehensive overview. The results are summarized in Table 1 by year of inclusion (2018 vs. 2022), assessing changes before and after the implementation of the secondary prevention program.

A total of 409 patients with acute coronary syndrome (ACS) were included in the study: 200 in the pre-intervention cohort (2018) and 209 in the post-intervention cohort (2022). Of these, 279 (68.2%) were men, and 130 (31.8%) were women. The proportion of female patients increased modestly following the implementation of the program.

The median age was 70.5 years in 2018 and 69 years in 2022, with a statistically significant difference. Consistent with prior evidence, women were significantly older (mean 74.3 ± 8.7 years vs. 64.8 ± 9.3 in men). No significant differences were found between periods in most comorbidities, except for dyslipidemia, which was significantly more frequent in 2018. Active smoking was more prevalent among men in both cohorts, and women had a higher prevalence of diabetes (51% vs. 32%) and obesity (39% vs. 26%).

There were no significant differences between years in the prevalence of diabetes, hypertension, obesity, chronic kidney disease, atrial fibrillation, or sleep apnea. The prevalence of previous coronary events was slightly higher in the post-program group for both sexes and higher in men in both periods, although these differences did not reach statistical significance.

Regarding ACS type, no sex-based differences were observed in either year.

### 3.2. Analytical Monitoring

A major improvement in post-discharge monitoring was observed after program implementation. In 2018, approximately half of patients lacked any blood test during the first four months after discharge (50.8% men, 54.0% women). By 2022, this fell dramatically to 8.7% in men and 16.4% in women (*p* < 0.001 for both). This improvement reflects the impact of systematic nurse-led follow-up, ensuring that nearly all patients were clinically reviewed within early recovery.

### 3.3. Cardiovascular Risk Factors Control

#### 3.3.1. Early Follow-Up (1–4 Months)

Both sexes showed significant improvement in lipid control, particularly men. The proportion achieving LDL-c < 55 mg/dL rose from 10.6% to 39.4% in men (*p* < 0.001) and from 4.0% to 23.9% in women (*p* = 0.003). Glycemic control (HbA1c < 7%) improved significantly in men (24.5% to 42.5%, *p* < 0.01) but not in women (20.5% to 23.9%, NS). Consequently, men achieved better control in early follow-up (LDL-c < 55 mg/dL: 39.4% vs. 23.9%, *p* = 0.03).

#### 3.3.2. Late Follow-Up (9–18 Months)

In the second analytic period (9–18 months), significant improvements in lipid and glycemic control were observed in both men and women, with higher rates of control in 2022 compared to 2018. For example, the proportion of men achieving STEP 2 lipid control increased from 9.8% to 37.8% (*p* < 0.001), and for women, it increased from 10.4% to 39.1% (*p* < 0.001). Unlike the early follow-up period, no significant sex differences were observed in lipid or glycemic control at 9–18 months. This pattern suggests that the program’s sustained, structured approach equalized outcomes over time, reducing early sex disparities.

#### 3.3.3. Predictors of Intensified Lipid Control

In multivariable logistic regression, the only variable independently associated with achieving STEP 2 lipid control belonged to the post-intervention cohort (OR 4.12, 95% CI 2.5–6.7, *p* < 0.001), in both men and women. Neither age nor sex independently predicted control after adjustment.

### 3.4. Mortality

At 18 months post-discharge, mortality decreased in both sexes following program implementation. In men, mortality declined from 9.1% in 2018 to 6.6% in 2022 (relative reduction of 27.5%), and in women, mortality fell from 10.5 to 5.5% in 2022 (relative reduction of 47.6%). Although sex-stratified Cox analyses did not reach statistical significance individually (HR men 0.71, *p* = 0.43; HR women 0.51, *p* = 0.30), the difference in relative reduction between sexes was significant (*p* = 0.0001), favoring women.

**Figure 2:**
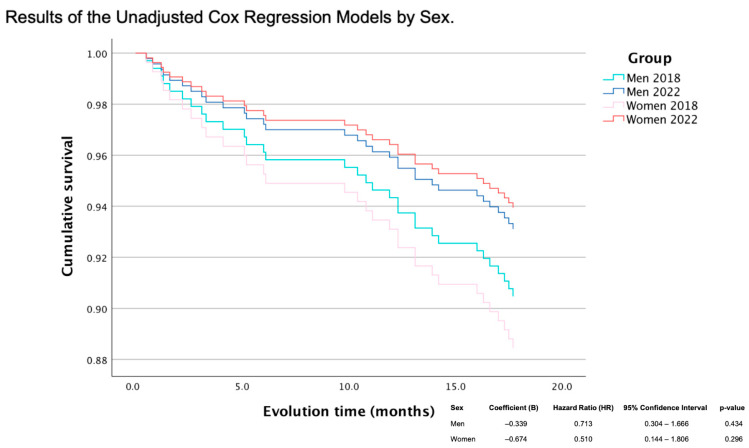
Cumulative survival by sex.

The cumulative survival curves illustrated improved survival in both sexes post-program, with the steeper slope reduction in women suggesting a greater benefit once equity in access was achieved (HR men: 0.713, *p* = 0.434; HR women: 0.510, *p* = 0.296) (Figure 2).

## 4. Discussion

This study demonstrates that a nurse-led, systematically inclusive secondary prevention program can simultaneously improve clinical outcomes and reduce gender-based disparities after ACS.

### 4.1. Nurse-Led Coordination and the Role of the APN

Traditional cardiac rehabilitation models often rely on selective referral or self-enrollment, perpetuating sex-based inequities [16,17]. By replacing referral with automatic inclusion, this program ensured that women—who historically experience lower referral and participation rates—received the same intensity of follow-up as men, effectively narrowing the gender gap in post-ACS prognosis. The advanced practice nurse (APN) played a pivotal role in this model, coordinating multidisciplinary care and ensuring that the management of clinical factors was integrated with an intensive focus on psychosocial factors. This coordination is critical because psychiatric comorbidities—particularly depression and anxiety—are more prevalent among women and act as independent predictors of adverse events [18,19,20], and these elements can affect participation in rehabilitation and long-term implementation of lifestyle measures.

By utilizing systematic monitoring and a patient-centered perspective, the APN helps overcome gender-related barriers, such as the persistent underestimation of cardiovascular risk in women and the lower likelihood of receiving intensified therapies. Ultimately, this model transforms evidence into equitable care by ensuring the multifactorial interplay of baseline risk, system-level delays, systemic inequities, and psychosocial factors through a single, coordinated pathway, combining clinical expertise, continuity, and a patient-centered perspective.

### 4.2. Bridging the Gender Gap

Initial disparities in lipid and glycemic control favored men during early follow-up, likely reflecting pre-existing differences in treatment intensity or adherence at discharge time [21,22]. However, by 18 months, outcomes were equivalent between sexes, suggesting that structured, sustained follow-up neutralized early disadvantages for women. As highlighted by recent reviews, sex-based inequalities in cardiovascular health are often driven by systemic and healthcare-related factors [16]. Women and older adults, in particular, tend to have more frequent contact with primary care services. When these services are effectively integrated with hospital-based programs, as was the case in this intervention, they may serve as key drivers for equity in long-term cardiovascular care [23].

Importantly, women exhibited a larger relative reduction in mortality (47.6%). Consistent with prior evidence [24,25], our pre-intervention cohort showed a higher mortality rate among women following an acute coronary syndrome. The greater relative reduction in cardiovascular mortality observed in women may be partly explained by their worse baseline prognosis, where a higher starting mortality increases the potential relative impact of an intervention. While a limitation of this study is the lack of data on pharmacological adherence or participation in therapeutic exercise, the program began systematically including therapeutic exercise in April 2022, with referral rates exceeding 55% in both sexes. The documented benefits of exercise in post-ACS could also have contributed to the greater mortality reduction observed among women [26,27]. Emerging evidence suggests that physical training may yield proportionally greater cardiovascular and non-cardiovascular mortality benefits in women [28,29]. The combination of exercise-based rehabilitation and continuous nurse-led support may explain the greater mortality reduction observed among women.

Beyond traditional metabolic risk factors, the differential prognosis in women must be framed within the emerging concept of the exposome—the cumulative measure of environmental influences and associated biological responses throughout the life course. Recent evidence suggests that women may be more susceptible to non-classic coronary risk factors, such as air pollution and environmental noise, which act as chronic stressors [30]. Fine particulate matter and noise pollution have been linked to sustained sympathetic activation and systemic inflammation, pathways that may be more reactive in the female cardiovascular system [31]. Furthermore, the psychological component of the exposome—encompassing depression, anxiety, and socio-environmental stress—often manifests differently across genders. The intersectionality of these factors—where environmental stressors like noise or pollution coexist with higher rates of psychological distress—creates a synergistic, detrimental effect on the female heart. Addressing the gender gap in ACS, therefore, requires a holistic approach that accounts for this external exposome, moving beyond the clinic to consider the broader environmental and psychosocial context of the patient.

### 4.3. Strengths and Limitations

The study’s strengths include the use of real-world data from both hospital and primary care, enabling thorough long-term follow-up and sex-stratified analysis for assessing equity. Excluding in-hospital deaths allowed for a clearer focus on secondary prevention outcomes. However, the retrospective pre–post observational design inherently precludes definitive causal inference, as the findings represent temporal associations rather than direct causality. While the positive outcomes align with the implementation of the nurse-led program, we cannot dismiss the potential contribution of contemporaneous advancements in acute coronary syndrome (ACS) management between 2018 and 2022. During this period, the increased clinical adoption of high-intensity statins, SGLT2 inhibitors, and PCSK9 inhibitors may have independently influenced the observed prognostic trends. The study also focused mainly on LDL cholesterol and HbA1c, omitting other important factors like blood pressure, medication adherence, and lifestyle behaviors. Nonetheless, the consistency of improvements across all metrics reinforces the robustness of the findings.

Baseline differences between men and women (e.g., age, diabetes, obesity, and comorbidity burden) may confound the observed association between the intervention period and mortality outcomes. In addition, the number of cardiovascular deaths over 18 months was limited, precluding robust multivariable adjustment and reducing statistical power; therefore, mortality comparisons when analyzed separately by sex relied primarily on Kaplan–Meier analyses and unadjusted Cox models. Nevertheless, the difference in relative mortality reduction between men (27.5%) and women (47.6%) was statistically significant, favoring women (*p* = 0.0001). The supervised therapeutic exercise component was implemented systematically from April 2022 for patients without significant physical limitations or contraindications. However, detailed data on attendance/adherence to exercise sessions and adherence to pharmacological therapy were not available and could not be analyzed.

This study was conducted at a single tertiary referral center, which may limit the generalizability of the findings. However, the evaluated intervention—a structured nurse-led secondary prevention program after acute coronary syndrome—is based on widely applicable organizational and clinical principles, including early follow-up, protocolized risk factor control, and multidisciplinary coordination, which are consistent with current international guideline recommendations. Moreover, the patient population and standard of care in our center are representative of other tertiary hospitals within the Spanish public healthcare system and similar European healthcare settings. We believe that the benefits observed in our study could be replicated in other tertiary centers in Spain and across Europe, particularly in those implementing a structured nurse-led secondary prevention program comparable to ours.

### 4.4. Systemic Implications

These findings extend beyond individual outcomes. They suggest that the gender gap in cardiovascular health is not fixed, but modifiable through structural design and nursing-led coordination. By embedding equity into the program’s core—universal inclusion, coordination, and proactive management—the initiative redefined secondary prevention from a selective intervention to a population-wide equity strategy.

## 5. Conclusions

A nurse-led secondary prevention program with systematic inclusion after ACS improved cardiovascular risk factor control and survival while significantly narrowing the gender gap in outcomes.

Women experienced a nearly 50% reduction in 18-month mortality, demonstrating that when access to structured, equitable care is guaranteed, the historical disadvantage of women in cardiovascular recovery can be reversed.

This study highlights the critical role of advanced nursing leadership in operationalizing equity, translating guidelines into practice, and ensuring sustained, data-driven follow-up. Implementing similar models could be a pivotal step toward closing the gender gap in cardiovascular prevention and outcomes.

## Figures and Tables

**Table 1:** Sociodemographic and clinical characteristics and degree of control of CVRF in men and women with ACS before (2018) and after (2022) the implementation of the secondary prevention program.

	Before Implementation (2018)			Post Implementation (2022)		
	Men n = 143	Women n = 57	*p*	Men n = 136	Women n = 73	*p*
**Age, years (median [IQR])**	70 [57–77]	76 [67–84]	<0.001	66 [56–75.8]	74 [64–82.5]	<0.001
**Comorbidities**						
Diabetes Mellitus, n (%)	40 (30.3)	24 (45.3)	0.053	37 (30.3)	25 (36.2)	0.403
Hypertension, n (%)	97 (73.5)	39 (73.6)	0.989	80 (65.6)	53 (76.8)	0.105
Hyperlipidemia, n (%)	98 (74.2)	37 (69.8)	0.539	74 (60.7)	36 (52.2)	0.255
Smoking, n (%)	43 (32.6)	7 (13.2)	0.007	45 (36.9)	13 (18.8)	0.009
Obesity, n (%)	23 (17.4)	15 (28.3)	0.098	24 (19.7)	20 (29.0)	0.142
CKD, n (%)	8 (6.1)	3 (5.7)	0.999	8 (6.6)	2 (2.9)	0.334
AF/Flutter, n (%)	2 (1.5)	4 (7.5)	0.057	2 (1.6)	0 (0.0)	0.536
Previous coronary event, n (%)	8 (6.1)	1 (1.9)	0.451	13 (10.7)	2 (2.9)	0.054
**Type of AMI, n (%)**			0.736			0.918
STEMI	69 (48.3)	26 (45.6)		57 (41.9)	35 (47.9)	
NSTEMI	74 (51.7)	31 (54.4)		76 (55.9)	34 (46.6)	
Others	0 (0.0)	0 (0.0)		3 (2.2)	4 (5.5)	
						
**1–4 month control (n)**	132	50		127	67	
Blood test performed, n (%)	65 (49.2)	23 (46.0)	<0.001	116 (91.3)	56 (83.5)	<0.001
LDL-c < 55 mg/dL, n (%)	14 (10.6)	2 (4.0)	<0.001	50 (39.4)	16 (23.9)	0.003
LDL-c < 70 mg/dL, n (%)	19 (14.4)	6 (12.0)	<0.001	75 (59.1)	26 (38.8)	0.001
non-HDL cholesterol < 85 mg/dL, n (%)	17 (12.9)	3 (6.0)	0.025	30 (23.6)	11 (16.4)	0.086
non-HDL cholesterol < 100 mg/dL, n (%)	21 (15.9)	6 (12.0)	0.005	39 (30.7)	14 (20.9)	0.206
STEP 2 control, n (%)	15 (11.4)	2 (4.0)	<0.001	53 (41.7)	17 (25.4)	0.002
STEP 1 control, n (%)	21 (15.9)	6 (12.0)	<0.001	78 (61.4)	27 (40.3)	<0.001
HbA1c < 7%, n (%)	16 (12.1)	9 (18.0)	<0.001	54 (42.5)	16 (23.9)	0.443
						
**9–18 month control (n)**	122	48		119	64	
Blood test performed, n (%)	88 (72.1)	36 (75.0)	<0.001	109 (91.5)	61 (95.3)	0.001
LDL-c < 55 mg/dL, n (%)	11 (9.0)	5 (10.4)	<0.001	45 (37.8)	16 (25.0)	0.050
LDL-c < 70 mg/dL, n (%)	21 (17.5)	8 (16.7)	<0.001	66 (55.5)	27 (42.2)	0.004
non-HDL cholesterol < 85 mg/dL, n (%)	14 (11.5)	5 (10.4)	<0.001	39 (32.8)	16 (25.0)	0.050
non-HDL cholesterol < 100 mg/dL, n (%)	16 (13.1)	8 (16.7)	<0.001	47 (39.5)	22 (34.4)	0.036
STEP 2 control, n (%)	12 (9.8)	5 (10.4)	<0.001	45 (37.8)	25 (39.1)	<0.001
STEP 1 control, n (%)	22 (18.0)	6 (12.5)	<0.001	60 (50.4)	33 (51.6)	<0.001
HbA1c < 7%, n (%)	10 (8.2)	6 (12.5)	<0.001	48 (40.3)	26 (40.6)	0.001

Note: Patients without LDL-c or HbA1c data were considered not controlled. STEP 1 lipid control: LDL-c < 70 mg/dL and/or non-HDL cholesterol < 100 mg/dL. STEP 2 lipid control LDL-c < 55 mg/dL and/or non-HDL cholesterol < 85 mg/dL. Abbreviations: CKD: chronic kidney disease. LDL-c: low-density lipoprotein cholesterol; HDL: high-density lipoprotein; HbA1c: glycated hemoglobin.

## Data Availability

The original contributions presented in this study are included in the article. Further inquiries can be directed to the corresponding author(s).
